# Neutral and Pectic Heteropolysaccharides Isolated from *Opuntia joconostle* Mucilage: Composition, Molecular Dimensions and Prebiotic Potential

**DOI:** 10.3390/ijms24043208

**Published:** 2023-02-06

**Authors:** José Manuel Cruz-Rubio, Alessandra Riva, Justyna Cybulska, Artur Zdunek, David Berry, Renate Loeppert, Helmut Viernstein, Werner Praznik, Fatemeh Maghuly

**Affiliations:** 1 Plant Functional Genomics, Institute of Molecular Biotechnology, Department of Biotechnology, BOKU-VIBT, University of Natural Resources and Life Sciences Vienna, Muthgasse 18, 1190 Vienna, Austria; 2 Department of Pharmaceutical Sciences, Division of Pharmaceutical Technology and Biopharmaceutics, University of Vienna, Josef Holaubek Platz 2, 1090 Vienna, Austria; 3 Centre for Microbiology and Environmental Systems Science, Department of Microbiology and Ecosystem Science, Division of Microbial Ecology, University of Vienna, 1090 Vienna, Austria; 4 Institute of Agrophysics, Polish Academy of Sciences, Doświadczalna 4, 20-290 Lublin, Poland

**Keywords:** SEC, ATR FT IR, HPAEC-PAD, methanolysis, growth curves, heteropolysaccharides, mucilage, *Opuntia* spp., mucilage, cactus, nopal

## Abstract

*Opuntia joconostle* is a semi-wild cactus cultivated for its fruit. However, the cladodes are often discarded, wasting the potentially useful mucilage in them. The mucilage is composed primarily of heteropolysaccharides, characterized by their molar mass distribution, monosaccharide composition, structural features (by vibrational spectroscopy, FT IR, and atomic force microscopy, AFM), and fermentability by known saccharolytic commensal members of the gut microbiota. After fractionation with ion exchange chromatography, four polysaccharides were found: one neutral (composed mainly of galactose, arabinose, and xylose) and three acidic, with a galacturonic acid content from 10 to 35%_mol_. Their average molar masses ranged from 1.8 × 10^5^ to 2.8 × 10^5^ g·mol^−1^. Distinct structural features such as galactan, arabinan, xylan, and galacturonan motifs were present in the FT IR spectra. The intra- and intermolecular interactions of the polysaccharides, and their effect on the aggregation behavior, were shown by AFM. The composition and structural features of these polysaccharides were reflected in their prebiotic potential. *Lactobacilli* and *Bifidobacteria* were not able to utilize them, whereas members of *Bacteroidetes* showed utilization capacity. The obtained data suggest a high economic potential for this *Opuntia* species, with potential uses such as animal feed in arid areas, precise prebiotic, and symbiotic formulations, or as the carbon skeleton source in a green refinery. Our methodology can be used to evaluate the saccharides as the phenotype of interest, helping to guide the breeding strategy.

## 1. Introduction

Hemicelluloses and pectins constitute one of the most complex molecules in plants, given the number of possible combinations between the composing monosaccharides and their glycosidic linkages. Their importance for the plant’s success in navigating the different environmental and biotic stresses present in their environment cannot be overstated. Furthermore, they are responsible for properties such as resistance to pathogen attack, firmness, and other organoleptic among other desirable properties. Thus, the amount of research dedicated to these polysaccharides is not surprising, including understanding their physical properties in their molecular environment and interactions with other cell wall components such as cellulose and lignin. New cultivars, achieved through conventional breeding, have modified and improved organoleptic traits valued by the consumers (e.g., sweetness, viscosity, etc.); however, it has only been done empirically.

Mucilage, a hydrocolloid composed majorly of heteropolysaccharides, is a key component responsible for the traits mentioned above. These heteropolysaccharides are not dissimilar in composition to the hemicelluloses and pectins of the cell wall. Mucilage is present in several botanical orders (e.g., Caryophyllales, Fabales, Malvales, Asparagales, Brassicales) [1,2], often in the seed coat or the roots. In these tissues, it is relatively straight forward to identify and isolate the mucilage, as it exudes in the presence of water, forming a viscous layer that can help retain moisture, immobilize nutrients, create a beneficial rhizosphere, or counteract biotic attacks, among others [3]. However, when mucilage is present in vegetative tissues, it is often not readily apparent, and it is often grouped with the polysaccharides that form the primary cell wall [4]. In specific plant families (e.g., Cactaceae, Annonaceae, Moraceae), the mucilage is stored in specialized secretory cells called mucilage cells [5]. While this facilitates the identification and isolation of the mucilage, the question of its role in planta becomes more complicated, as the large size of the mucilage hinders its mobility in the tissue. Among its proposed functions are drought [6] and frost [7] resistance and/or as a carbohydrate reserve [2].

In *Opuntia* spp., the mucilage is located in the seeds [8] and in mucilage cells present in the cladodes [9,10], where it is one of the main components of the water-soluble materials [11]. Furthermore, there is evidence that species with different degrees of domestication have different dietary fiber (i.e., polysaccharides) profiles [12,13]. Rhamnose, arabinose, xylose, galactose, and galacturonic acid (GalA) are the monosaccharides in the mucilage of two *Opuntia* species: *Opuntia ficus-indica* (domesticated) and *Opuntia joconostle* (semi-wild). Their main differences lie in the higher amount of arabinose, lower amount of galacturonic acid, higher average molar mass, and higher dispersity in *O. joconostle* [14]. Further research into the mucilage of these two species has shown that they form a supramolecular structure composed of covalently and non-covalently linked hetero oligo- and polysaccharides with very different molecular dimensions and monosaccharide compositions [15].

**Figure 1:**
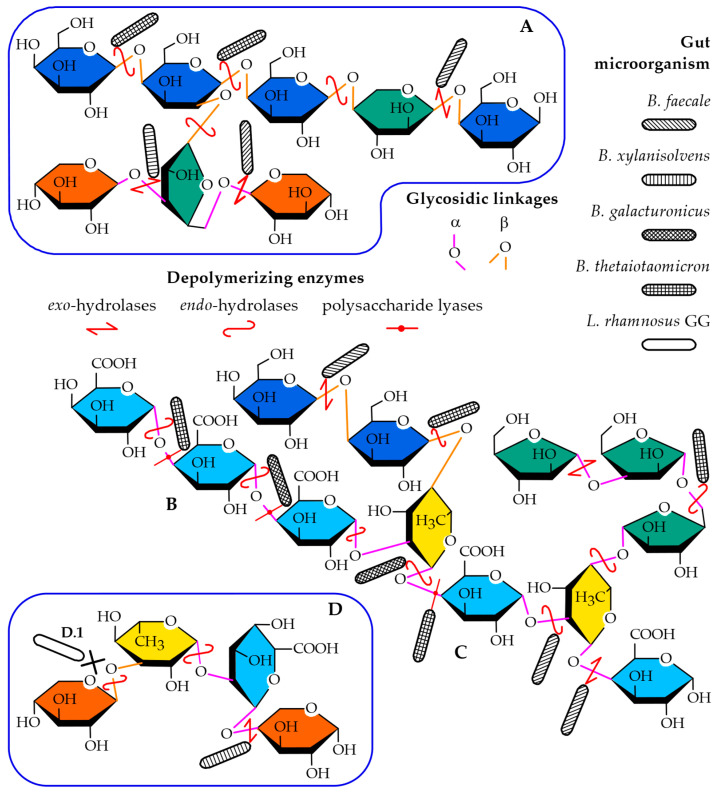
Common saccharide motifs exemplifying some of the monosaccharides and glycosidic linkages present in the mucilage of dicots. (**A**) Branched galactan (including β-Gal*p*-(1,4)-, β-Gal*p*-(1,2,4)-, β-Ara*p*-(1,4)-, α-Ara*f*-(1,3,5)-, and β-Xyl*p*-(t)). (**B**) Homogalacturonan (including α-GalA*p*-(1,4)-). (**C**) Rhamnogalacturonan I (including α-Rha*p*-(1,2,5)-, β-Gal*p*-(t), α-Ara*f*-(1,5)-, α-Ara*f*-(t), and α-GalA*p-*(t)). (**D**) Branches of xylan (including β-Xyl*p*-(1,3)-, α-Rha*p*-(1,3)-, α-GalA*p*-(1,2)-, and α-Xyl*p*-(t)). (D.1) representation of a linkage sterically unavailable for a microorganism. Galactose, blue; galacturonic acid, light blue; xylose, orange; rhamnose, yellow and arabinose, green. α-linkages are represented in pink, while β-linkages are colored orange. Depolymerizing enzymes are drawn as red lines: *endo*-acting hydrolases as rounded double arrows, *exo*-acting hydrolases as double arrows, and lyases as lines with a dot in the middle. The different bacteria (*B. faecales*, rod with diagonal fill, *B. xylanisolvens*, rod with vertical fill, *B. thetaiotaomicron*, rod with square fill, *B. galacturonicus* rod with crosshatch fill, *L. rhamnosus* GG, no fill) are shown attached to the red hydrolases in potential sites of depolymerization, based on their reported hydrolytic toolkit.

To gain further insights into the composition and structure-prebiotic potential relationship of the mucilage, several questions must be answered. These include finding the monosaccharides and how they are arranged in the polysaccharides that form the mucilage and investigating the covalent and non-covalent interactions between the polysaccharides (both intra- and inter-molecularly) and water. Finally, the combination of the factors listed above determines the availability of the glycosidic linkages to the polysaccharide-degrading enzymes used by the probiotic bacteria. Figure 1 shows common (and representative) motifs found in the polysaccharides that form mucilages and cell walls of dicots. It also shows how bacteria might depolymerize the saccharides either by removing the monosaccharides located in a reducing end (*exo*-hydrolases) or by hydrolyzing glycosidic bonds in the main chain/branching points (*endo*-hydrolases) (Figure 1A) or by a β-elimination mechanism in homogalacturonan (Figure 1B) and rhamnogalacturonan I (Figure 1C) regions (lyases). Furthermore, the effects on the polysaccharides capacity to serve as a substrate for the enzymes given their anomeric configuration (α- or β) or linkage position (e.g., 1,4; 1,6; 1,2,4; 1,5) was investigated, in addition to their possible effect in the conformation of the polysaccharide in solution, which might then sterically hinder the active site of the enzyme (Figure 1D.1). 

Furthermore, from a human intake point of view, these polysaccharides support the growth of different groups of probiotic bacteria, modifying their effect on human health and nutrition [16]. Thus, if a rational breeding program is to be established, measurable phenotypic markers (such as the content or composition of the mucilage in the plant) need to be established. The current study delves deeper into the composition and prebiotic properties of the heteropolysaccharides that form the mucilage of *O. joconostle* cladodes and demonstrates that the mucilage is a supramolecular structure composed of complex neutral and acidic heteropolysaccharides. In addition, our work found that these polysaccharides were not utilized by the typically known probiotic bacteria (e.g., Lactobacilli and Bifidobacteria) but only by primary degraders and commensal members of the gut microbiota.

## 2. Results and Discussion

### 2.1. Mucilage Polysaccharides Fractionation

Our previous work demonstrated the existence of several oligo- and poly-saccharide populations in the mucilage. In addition, the relatively large amount of galacturonic acid [15] makes the mucilage a prime candidate for a charged-based separation. To this effect, a weak anionic exchanger was chosen for the separation (ion exchange chromatography, IEC). Ammonium formate was chosen as the eluent for the monovalent nature of its ions (removing potential ionic bridging artefacts in subsequent analysis), as well because the salt remains in solution after the methanol precipitation step, and any traces left in the solution would be removed during lyophilization. We found that an eluent strength higher than 1.0 M did not elute any more polysaccharides, and the recovery (defined as mass of resulting fractions/injected mucilage mass) was quite acceptable (>90%). The separation yielded between 75–85% of polysaccharides. At first, the recovery was assumed to be low, but it was found that the difference was due mostly to the presence of oligosaccharides and exchanged non-volatile salts, which co-eluted with the neutral fraction, but were washed away during the methanol precipitation step. Treatment of this neutral fraction with α-amylases did not release any noticeable amount of glucose, confirming the absence of α-linked glucans (e.g., starch). Most of the low amount of glucose in the mucilage comes from free glucose (and sucrose), as shown in [15], and confirmed by thin layer chromatography. Based on test runs, four eluent strengths (5 mM, 0.2, 0.4, and 1.0 M) gave a satisfactory separation. Galacturonic acid content increased as eluent strength increased. The resulting polysaccharides were designated as follows: OXO N (5 mM fraction), OXO 0.2 (0.2 M fraction), OXO 0.4 (0.4 M fraction), and OXO 1.0 (1.0 M fraction). Table 1 shows that the bulk of the polysaccharides corresponded to OXO 0.2 and OXO 0.4 fractions (ca. 80%*_w_*_/*w*_), making OXO N and OXO 1.0 minor components of the mucilage.

### 2.2. Molecular Dimensions

The mucilage (OXO M) has the largest molar mass as the sum of all the polysaccharides (Table 1). Given the lack of specificity in the non-covalent bonds that hold together the mucilage, it is also not surprising that it has a large dispersity. The NaCl eluent chosen for the size exclusion chromatography (SEC) separation minimizes the electrostatic interaction between the polysaccharides and the chromatographic resin. However, as an unbuffered system at a pH close to neutral, the galacturonosyl acid residues are present in different dissociation states, influenced by their p*K*_a_ and the residues in their vicinity. Therefore, some carboxyl groups could be ionized and free to interact via ionic bridges, while others can only interact via hydrogen bonds or van der Waals forces. As the content of galacturonic acid residues increase, their apparent hydrodynamic value is more affected by these phenomena, increasing the dispersity (Table 1) of the samples (OXO 0.2 < OXO 0.4 < OXO 1.0) as an artefact of the analysis conditions.

**Figure 2:**
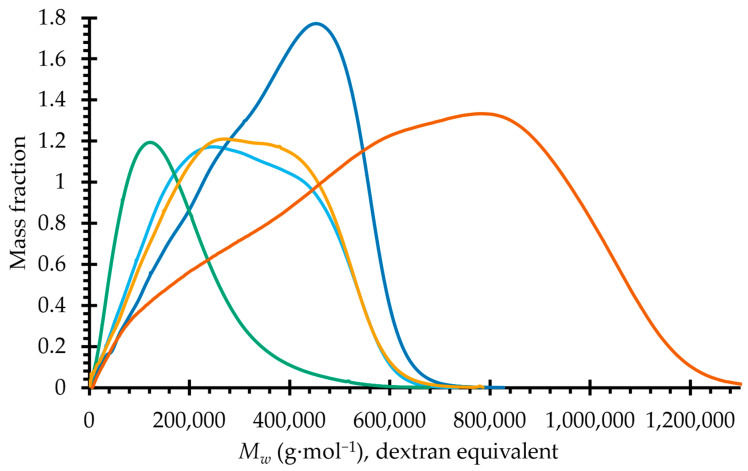
Molar mass distribution of the mucilage of *O. joconostle* and the polysaccharides found after IEC fractionation. Orange, OXO M; Sky blue, OXO N; Blue, OXO 0.2; Green, OXO 0.4; Yellow, OXO 1.0.

The molar mass distributions of the IEC-separated samples are shown in Figure 2. The sample with the smallest average molar mass is OXO 0.4, while the other three fractions share relatively similar molar mass profiles. The profile of OXO M was reported in our previous work [14].

### 2.3. Monosaccharide Composition and Content

All the IEC-separated fractions are composed of the same six monosaccharides: rhamnose, arabinose, xylose, galactose, fucose, and galacturonic acid (Table 2). However, their proportions are quite varied. OXO M does not contain fucose, while all their composing polysaccharides do. This can be explained as a result of a change in the hydrolysis method. OXO M [14] was hydrolyzed using only trifluoroacetic acid (TFA). Fucose is a labile sugar and was most likely degraded, as it was liberated from the polysaccharide. In the current method, the methanolysis step (in addition to cleaving the polysaccharide) forms methyl glycosides, which protects the released monosaccharides from further attack. The methylated monosaccharides return to their original form during the second hydrolysis step with TFA. The rhamnose level is constant for all the IEC samples at less than 10%_mol_. OXO N does not contain uronic acid, with galactose being the main sugar. The acidic polysaccharides, in contrast, have a constant amount of rhamnose, and the rest of the neutral sugars (arabinose, xylose, and galactose) all decrease as the content of galacturonic acid increases.

### 2.4. Structural Features: Fourier-Transform Infrared Spectroscopy (FT IR) Spectroscopy—SEC-UV/VIS

The most notorious feature of the FT IR spectra is that OXO M has a very distinct spectrum to those of its composing polysaccharides, with broader, less defined bands. This was expected, given its higher complexity. Additionally, there are small but noticeable signs of band frequency shifting caused by the intramolecular bonding of the polysaccharides, which increases the vibrational constraints of the rings, hydroxyl groups (via hydrogen bonding), and rigidizes the glycosidic linkages [17,18].

**Figure 3:**
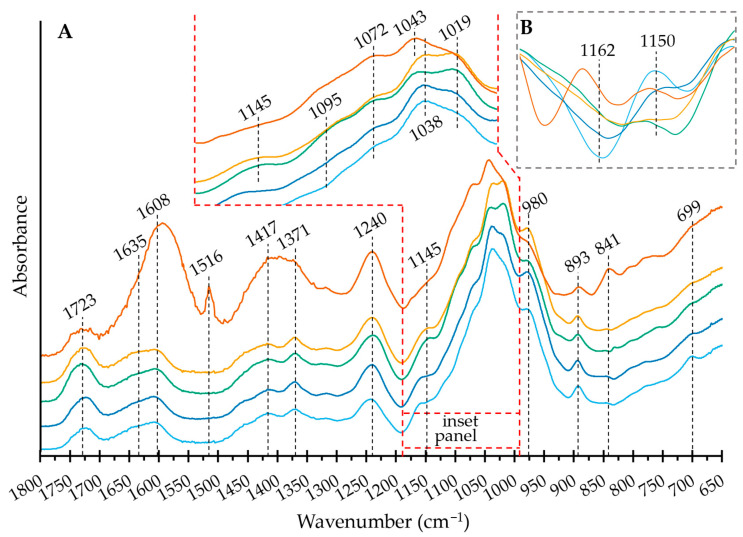
(**A**) FT IR spectra of the mucilage extracted from *O. joconostle* cladodes and its composing polysaccharides in the 1800–650 cm^−1^ range. OXO M (orange), OXO N (light blue), OXO 0.2 (dark blue), OXO 0.4 (green), OXO 1.0 (yellow). (**B**) Second derivative of the FT IR spectra (1132–1180 cm^−1^ range). Refer to Section 2.4. in the text for the band assignments.

**Figure A1:**
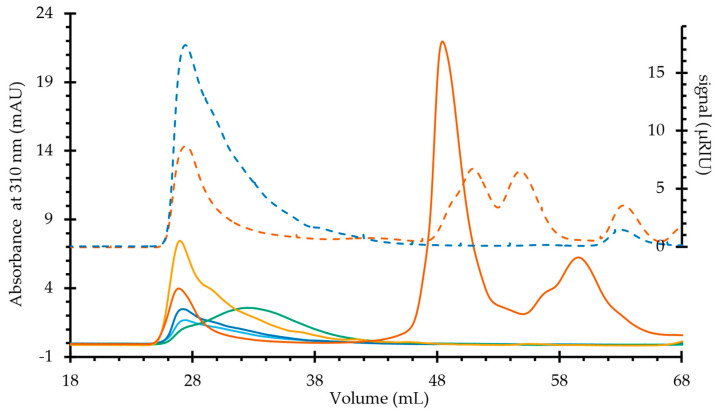
Size exclusion chromatogram showing the elution profile of the mucilage and IEC fractions monitored by absorption at 310 nm (solid lines), with the differential refractive index signal overlapped in broken lines. OXO M (orange), OXO N (light blue), OXO 0.2 (dark blue), OXO 0.4 (green), OXO 1.0 (yellow).

With this background, the band assignment and discussion will center on the IEC-separated heteropolysaccharides, as shown in Figure 3. The broad band at 1723 cm^−1^ has been assigned to esterified galacturonosyl, or in the case of the OXO N, galactosyl residues [19]. The band at 1608 cm^−1^ is assigned to the free carboxyl groups in pectins [20]. However, in this study, OXO M shows a much higher proportion of the first band than the second, in strong contrast to the IEC-separated samples, which show relatively similar areas for both bands. Absorbed water delivers a band at 1635 cm^−1^ [21], which obscures the contribution of the functional groups. The difference in intensity of this band to the IEC-separated samples serves as additional evidence of the higher amount of water fixed to the supramolecular assembly of the mucilage. Uronic acids also appear at this wave number, so this band likely results from the contributions of the water and uronic acids. In contrast to its components, OXO M has a prominent peak at 1516 cm^−1^. Palacio et al. have assigned this band to the aromatic C=C stretching of lignins and their phenolic backbone [22]. To corroborate this finding, the SEC analysis delivered in the UV/VIS plot at 310 nm a prominent peak at 48 mL without any corresponding signal in the RI plot (Figure A1). The wavelength of 310 nm was chosen as it is close to the absorption maximum of ferulic acid and its derivatives [23]. This peak is likely to be phenolics separated under the mild conditions of SEC. Additionally, a much smaller peak follows the shape of the polysaccharide peak (26–32 mL), indicating the covalently bound phenolics that help form the supramolecular structure. 

As a complementary example, the OXO 0.4 spectrum lacks the band at 1516 cm^−1^, with the SEC-UV/VIS profile showing a weak absorption peak of covalently bound phenolics following the broad polysaccharide profile (26–38 mL). Between 1480 and 1342 cm^−1^, OXO M shows a very broad band (Figure 3A). However, the IEC-separated samples separate this area into two better defined bands: one centered at 1371 cm^−1^ and one at 1417 cm^−1^. The former has been assigned to xyloglucans and/or cellulose [17,24]. However, the low glucose content (<2.5%_mol_) excludes this possibility. Thus, it is prudent to assign it only to the C–H vibrations and CH_2_ bending characteristic to hemicelluloses, while the band at 1417 could be ascribed to CH_2_ symmetric bending.

The unresolved band at 1132–1180 cm^−1^ (centered around 1145 cm^−1^) includes the O–C–O glycosidic bond vibrations [25]. Figure 3B shows the second derivative of the spectra in this region. As reported, the band at 1150 cm^−1^ cannot be totally ascribed to the glycosidic bonds in the pectic domains [18,25]. However, the band intensity decreases as the galacturonic acid content increases and could be used as a fingerprint region in addition to the band at 1162 cm^−1^. The bands at 1162 cm^−1^ show a relatively large intensity difference, which could be speculated to belong to the glycosidic linkages in the galactan domains, as the number of galactose moieties is the only remaining component with such a large variation. 

The region from 1132 to 990 cm^−1^ includes the non-localized, highly coupled vibrational modes of polysaccharide backbones [26]. It is dominated by an overlap of ring vibrations, the stretching vibration of (C–OH) side groups and the (C–O–C) glycosidic bond vibration [18]. The inset in Figure 3A shows this region in detail. The band at 1095 cm^−1^, with a slightly higher intensity for OXO 0.4 and OXO 1.0, can be explained by the increased levels of galacturonic acid in the samples compared to OXO N and OXO 0.2 [25]. The bands at 1072 and 1038 cm^−1^ are characteristic of β-linked galactan [18], while the peak at 1043 cm^−1^ (with a corresponding band broadening due to other types of linkages) can be assigned to the arabinosyl-galactosyl linked residues [18]. The band at 1019 cm^−1^, typical for pectins [17,18,25], behaves as expected, with the band intensity increasing with the galacturonic acid content of the sample. The band at 980 cm^−1^ has been assigned to xylans and arabinoxylans [25] and is consistent with the monosaccharide composition; OXO 0.2 (highest xylose content) has the highest intensity, while OXO N contains the least amount of xylose (Figure 3A). 

In the anomeric region, the band at 893 cm^−1^ indicates a β-configuration, while 841 cm^−1^ indicates the α-anomer form of the pyranoid ring [18]. In the present investigation, the IEC step breaks the supramolecular structure, allowing the precipitation steps to remove the low molar mass components. This includes the glucose and sucrose trapped by the mucilage structure, as described in [15]. Therefore, OXO M has a far more intense α-anomer band than the IEC separated samples, with an intense band at 893 cm^−1^ and a faint band at 841 cm^−1^ (Figure 3A). This confirms that most of the sugar moieties in the heteropolysaccharides are in the β-configuration, with arabinose as the potential exception. 

### 2.5. Structural Features: Atomic Force Microscopy (AFM) Imaging

**Figure 4:**
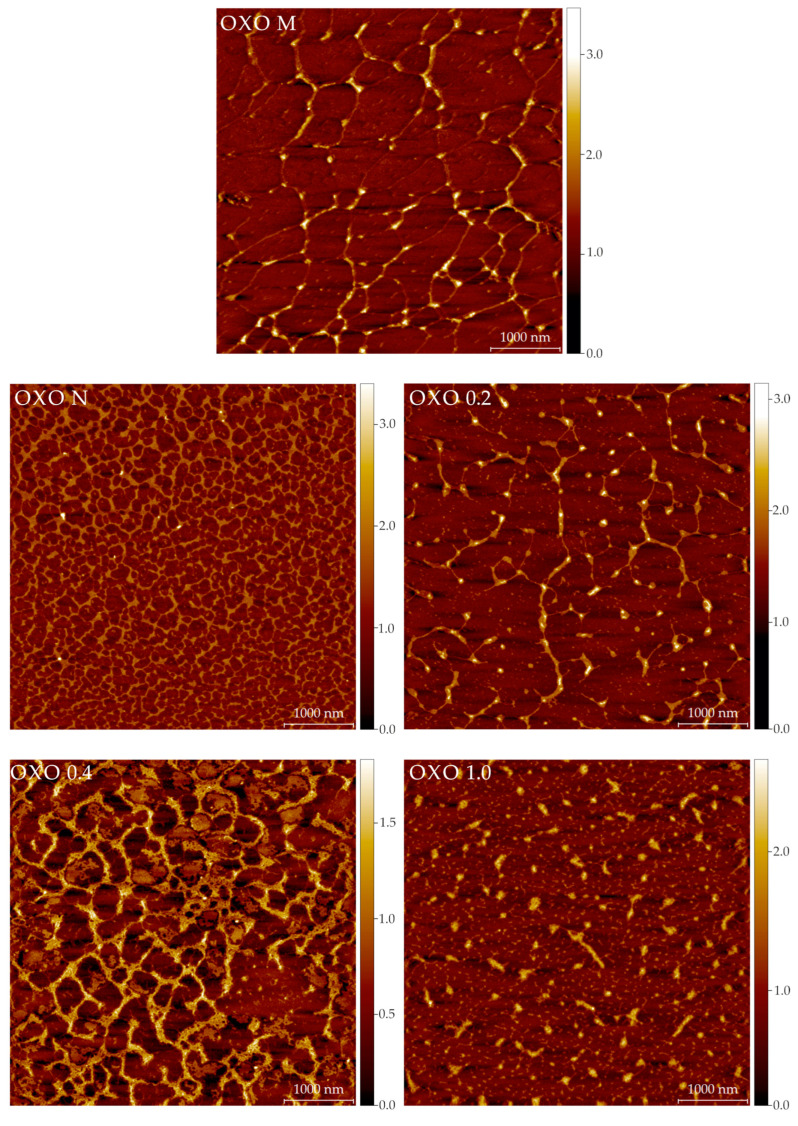
Atomic force microscopy representative images (5 × 5 μm) of the mucilage extracted from *O. joconostle* cladodes and its composing polysaccharides. The color scale on the right indicates the height in nm.

**Figure A2:**
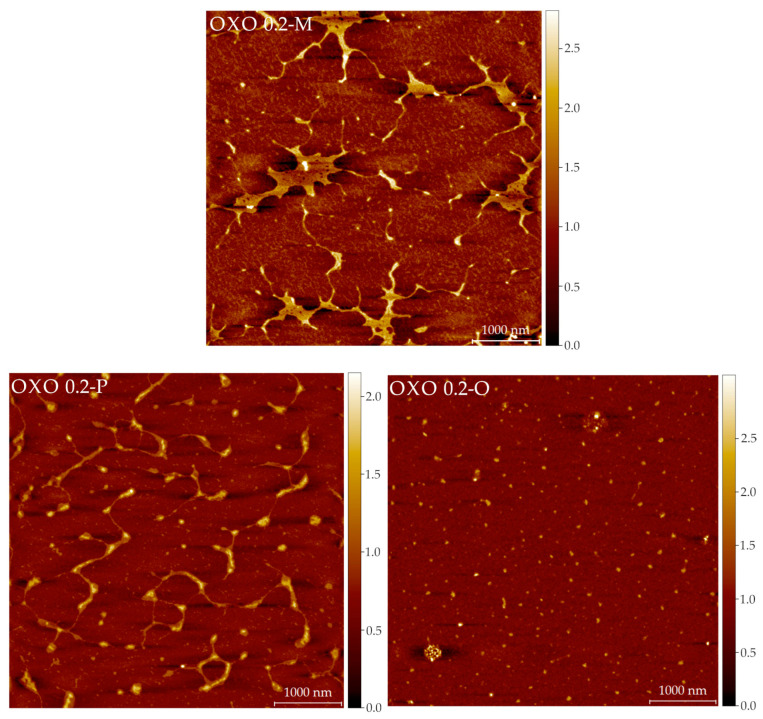
Representative images (5 × 5 μm) of the OXO 0.2 fractions after IEC but before SEC separation (**top**) and after SEC fractionation (**bottom left**, polysaccharide fraction, **bottom right**, low molar mass components). The color scale on the right indicates the height in nm.

Representative AFM images for all the samples are shown in Figure 4. Table A1 includes the calculated parameters (from all the images) for the polysaccharides. It is difficult to correlate the imaging results (Figure 4) with the SEC results (Table 1). In the SEC analyses, the polysaccharides are in a dilute, solvated state (meaning the interactions are primarely between water and the molecules). In AFM imaging, the samples are in a dry state, with molecules interacting with other molecules (as the concentration increases during the drying period) and the mica surface. The differences in these interactions allow us to speculate on the structures seen by AFM. AFM images show that molecules in all samples are prone to interlinking and aggregation during drying in mica. However, their final structures are distinct. OXO M forms fibers that are long, flat, and linked in 3 or 4 nodes. This could be evidence of the supramolecular structure in a minimal energy state, as most electrostatic interactions happen between molecules. The mean height of the OXO M fibers is relatively small (0.33 nm), but there are fragments that appear thicker. Moreover, most of the nodes have a greater height than the linear part of the network. In sharp contrast, OXO N forms a very tight, highly cross-linked network of macromolecules. In a solvated state, the hydrogen and van der Waals forces dominate the intramolecular interactions in this polysaccharide. As water is removed during the drying process, the molecules tend to self-aggregate without the interference of ionic interactions due to the lack of acidic moieties, as is evidenced in the AFM images. OXO 0.2 displays features similar to OXO M, but the network of OXO 0.2 is less dense and molecules are shorter than OXO M. This behaviour indicates an equilibrium between the electrostatic and ionic forces. Similarly to OXO N, OXO 0.4 displays a tight network; however, the size of the voids is considerably larger. As this fraction has a higher galacturonic acid content, the influence of the ionic residues may cause this behaviour upon drying. OXO 1.0 shows short molecules and small, rounder, and taller aggregates that could be considered evidence of the molecules self-aggregation, facilitated by the flexibility granted by the greater number of galacturonic acid moieties in the polysaccharide backbone. Finally, Figure A2 is used as an example of how all the components of the mucilage contribute to the overall shape and conformation of the polysaccharide. OXO 0.2-M correspond to the fraction after IEC separation. Large intramolecular aggregates can be seen. OXO 0.2-P is the same image as described before; long molecules with minimal intramolecular or self-aggregation. OXO 0.2-O includes the mono-, oligo-saccharides, and other low molar mass components separated during the preparative SEC step. Their size (39.8 ± 0.4 nm in diameter) is an order of magnitude smaller than the polysaccharides, but their importance cannot be overstated, as their interaction with the polysaccharides eventually forms the large structures seen in OXO 0.2 M (Figure A2).

### 2.6. Prebiotic Potential

**Figure 5:**
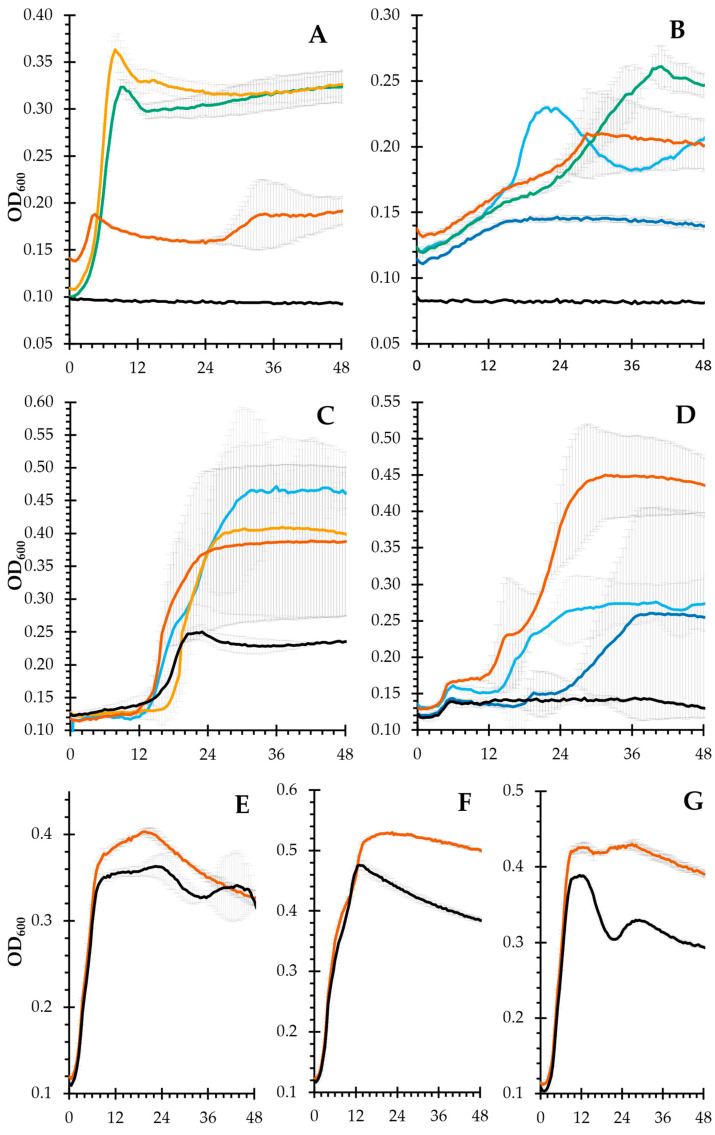
Growth curves of the tested intestinal bacteria. (**A**), *B. galacturonicus*; (**B**), *B. thetaiotaomicron*; (**C**), *B. xylanosolvens*; (**D**), *B. faecale*; (**E**), *L. rhamnosus* GG; (**F**), *L. plantarum*; (**G**), *B. longum*. For all panels: OXO M (orange), OXO N (light blue), OXO 0.2 (dark blue), OXO 0.4 (green), OXO 1.0 (yellow), negative control (black, NA). Compounds not shown did not induce growth for the corresponding bacteria. Error bars indicate standard error (n = 2).

Our previous results [15], indicating that the tested probiotics were not fully capable of fermenting the polysaccharides in the mucilage, led us to select members of the gut microbiota known for their ability to use complex carbohydrates as a carbon source. Among the tested strains, *Bacteroides galacturonicus* (Figure 5A) cannot metabolize glucose; therefore, it should use uronic acids. Its highest growth was reached with OXO 1.0 (GalA content, 35%_mol_), followed by OXO 0.4 (GalA content, 25%_mol_). Surprisingly, no growth was seen with OXO 0.2. This could be either due to the galacturonic acid moieties being linked in a form that made them unavailable to the microorganism, or that the galacturonic acid content was so low that the bacteria did not have enough time/energy to release enough galacturonic acid from the polysaccharide to start growing. *Bacteroides thetaiotaomicron*, with its saccharolytic capabilities (over 250 hydrolases, 80 transferases, 15 lyases, and 19 esterases), can use dozens of dietary plant polysaccharides [27]. Therefore, it was able to ferment all tested fractions. *B. xylanisolvens* (DSMZ 18836) can only utilize xylosyl-residues present in side chains, as reported in [28]. OXO 0.2 and OXO N were the only two IEC fractions that supported its growth (Figure 5C). The former has the largest amount of xylose, while the latter has the least amount of it. This indicates structural differences in the polysaccharides: OXO N and OXO 0.2 are related by having xylosyl residues in the side chains, while OXO 0.4 and OXO 1.0 have their xylosyl residues in a position not available to the microorganism. *B. faecale* (Figure 5D) was able to ferment OXO N and OXO 0.2, and in doing so, it is possible that it untangled the supramolecular structure, releasing the remaining glucose that boosted the growth seen in OXO M. 

Finally, it is worth noting that the bacteria typically considered probiotic [29] (in our case, *L. rhamnosus* GG, *L. plantarum*, and *B. longum*) could not utilize any of the polysaccharides and were only barely able to grow in the mucilage fortified media, with maximum growth values consistent with our previous results [14]. Therefore, this growth is caused by the small amounts of mono-, di-, and oligosaccharides found within the mucilage. 

## 3. Materials and Methods

### 3.1. Plant Material

Cladodes from *O. joconostle* F.A.C. Weber cultivated in Cuquío, Jalisco, Mexico (20°54′31.3″ N, 103°00′05.1″ W), were collected and transported to the laboratory, where the thorns and glochids were removed. Next, they were julienned into 3–4 mm thin strips, frozen at −80 °C, and lyophilized overnight. The dry material was stored at −80 °C until further use.

### 3.2. Chemicals, Carbohydrate Standards and Intestinal Bacterial Species

All chemicals and monosaccharide standards were at least of analytical grade and purchased from Sigma-Aldrich (St. Louis, MO, USA). The dextran *M_w_* narrow standards were supplied by Pharmacosmos A/S (Holbaek, Denmark). In addition, 50%*_w_*_/*v*_ sodium hydroxide extra pure solution for anion exchange chromatography eluent preparation was sourced from Acros Organics (Geel, Belgium). 

*Bacteroides galacturonicus* (DSM 3978) and *Bacteroides xylanisolvens* (DSM 18836) were sourced from the DSMZ (Braunschweig, Germany). *Lactobacillus rhamnosus* GG (ATCC 53103), *Bifidobacterium longum* subsp. *infantis* (ATCC 15697), and *Lactobacillus plantarum* subsp. *plantarum* (ATCC 14917) were sourced from the ATCC (Manassas, VA, USA). *Bacteroides thetaiotaomicron* and *Bifidobacterium faecale* were isolated from human stool samples, as described in [30].

### 3.3. Mucilage Extraction

The dry cladodes were processed in a kitchen blender until they passed a U.S. mesh 80 (165 μm). Non-polar compounds such as chlorophylls, lipids, waxes, and triterpenes were removed by extracting the dry cladode powder with acetone three times. Low molecular weight polar components (e.g., phenolics, mono-, di-, and oligosaccharides, and salts) were removed by extracting three times with 95%*_v_*_/*v*_ methanol. The mucilage was extracted by treating the purified cladodes with deionized water at 80 °C under intense mixing. Centrifugation followed, to separate the mucilage from the cladode solids, which were re-extracted twice more under the same conditions. The three water-soluble mucilage extracts were pooled, lyophilized, and stored in a desiccator until further use. The detailed purification and extraction method can be seen in [14]. This sample is designated as OXO M.

### 3.4. Mucilage Fractionation by Preparative Ion Exchange Chromatography

Ammonium formate buffers (AFB), pH 5.2, at concentrations of 4.0, 1.0, 0.4, 0.2, and 0.005 M, were prepared by measuring the calculated amount of formic acid to reach the desired molarity and bringing the pH to 5.2 by the addition of 32% ammonium hydroxide solution. A 25 × 300 mm glass Econo-Column (Bio-Rad Laboratories GmbH, Feldkirchen, Germany) was packed with Toyopearl DEAE 650-M (Tosoh Bioscience GmbH, Griesheim, Germany) to a height of 180 mm. The column was regenerated by passing 20 column volumes of 4.0 M AFB and conditioned for use by passing 10 column volumes of 5 mM AFB. 

Approximately 1.2 g of mucilage was dissolved overnight at room temperature in 16 mL of 5 mM AFB. This solution was slowly introduced into the column, and a stepwise elution was carried out by consecutively passing 86 mL of 5 mM, 0.2 M, 0.4 M, and 1.0 M AFB (each AFB strength was collected into 10 tubes). Each AFB elution strength was named OXO N, OXO 0.2, OXO 0.4, and OXO 1.0, respectively. Carbohydrate presence was evaluated by spotting 2 µL of the fraction in a thin layer chromatography plate strip and derivatizing with thymol-sulfuric reagent [14]. Next, the fractions showing carbohydrate presence were pooled, frozen, and lyophilized. To remove the ammonium formate and other residual salts, the lyophilized product was redissolved in 8 mL of deionized water, 80 mL of methanol was added, and the suspension was centrifugated at 12,000× *g* for 25 min. The supernatant was discarded, and the water/methanol wash step was repeated twice more. Finally, the precipitate was dissolved in 20 mL of water, frozen, lyophilized, and stored in a desiccator until further use.

### 3.5. Preparative Size Exclusion Chromatography

To remove all low molar mass components, the IEC-separated fractions were separated in a system consisting of a 15 × 360 mm precolumn packed with Toyopearl HW 40-S (Tosoh Bioscience GmbH), followed by 15 × 1070 mm Sephacryl S-200 HR and 15 × 1200 mm Sephacryl S-1000 SF columns (GE Healthcare Life Sciences, Uppsala, Sweden). The eluent was 50 mM AFB, pH 5.6 at a flow rate of 0.66 mL/min. Detection was achieved with a differential refractive index detector, and the system was calibrated using standard dextrans.

Ca. 120 mg of IEC fraction was dissolved in 1.5 mL of eluent and injected into the system. Salts and oligosaccharide containing fractions were discarded, and the lower molar mass peaks were separated from the high molar mass polysaccharide-containing fractions. Similar fractions were pooled together, frozen, and lyophilized twice in order to ensure the removal of the volatile salts.

### 3.6. Analytical Size Exclusion Chromatography

The molar mass distribution was evaluated by SEC with a system comprised of three 10 × 300 mm columns in series (Superose 6 → Superdex 200 → Superose 12, GE Healthcare Life Sciences, Uppsala, Sweden). The eluent was 0.5 M NaCl + 0.01 NaN_3_ at 0.6 mL∙min^−1^. A differential refractive index detector was used as the mass detector for the molar mass evaluation; absorbance at 310 nm was used to qualitatively evaluate the presence of ferulic acid and its derivatives. CPCWin 32 (a.h. group, Graz, Austria) was used to perform the calculations on the raw chromatographic data. The molar masses are reported as dextran equivalent.

### 3.7. Methanolysis—Trifluoroacetic Acid Hydrolysis

Approximately 2 mg of saccharide was dissolved in water to a final concentration of 2 mg∙mL^−1^. 20 µL aliquots of this solution were placed in the bottom of 13 × 100 mm glass test tubes and 400 µL of methanol was incorporated. The methanol was removed under reduced pressure, and this procedure was repeated two times. Next, 300 µL of 3 M anhydrous methanolic HCl with 0.3%*_v_*_/*v*_ 2,2-dimethoxypropane (as a water-scavenging agent) was added and the tubes were securely closed. Incubation under vigorous stirring at 80 °C was left to proceed for 72 h. After this time, the methanol and HCl were removed under reduced pressure at room temperature. 400 µL of methanol was added, stirred for 15 min, and evaporated under reduced pressure. This step was repeated four times to ensure total removal of the HCl. Afterwards, 150 µL of deionized water was added to the tube, stirred for 15 min, sonicated for 30 min, and stirred for 30 more minutes to ensure the total dilution of the resulting methyl glycosides. Next, 150 µL of 4 M TFA was added, and the tubes were closed and incubated at 120 °C for 2 h. After completion of the hydrolysis, the tubes were opened, 400 µL of methanol was added, and the contents evaporated under reduced pressure at room temperature. This step was repeated six times, after which neutral pH was achieved, signaling total removal of the TFA. Finally, 1 mL of 30%*_v_*_/*v*_ methanol was added to the tubes (resulting in a 1:50 dilution of the original sample) and stirred for 1 h. The solutions were transferred to Eppendorf tubes, centrifugated at 24,000× *g*, and the top 800 µL transferred to a chromatographic vial.

### 3.8. Monosaccharide Determination by High Performance Anion Exchange Chromatography—Pulsed Amperometric Detection (HPAEC-PAD)

The depolymerized samples were analyzed by HPAEC-PAD, as described in [14]. The monosaccharide mixture standards were treated under the same depolymerization conditions as the samples. The analyses were carried out in quadruplicate, and the confidence intervals were calculated using the *t*-value [31].

### 3.9. Total Carbohydrate Content

After evaluating the monosaccharide composition by HPAEC-PAD, standards closely matching the monosaccharide proportion of each sample were prepared at different concentration levels. These standards and the samples were analyzed via a semi-micro phenol-sulfuric method, as described in [14]. The analyses were carried out in triplicate and the confidence intervals were calculated using *t*-values [31].

### 3.10. FT IR Spectroscopy

The FT IR spectra were recorded in the 4000–650 cm^−1^ range using a Nicolet 6700 FT-IR (Thermo Scientific, Waltham, MA, USA) equipped with an attenuated total reflection (ATR) attachment. 200 scans per measurement were performed, and then averaged with a spectral resolution of 4 cm^−1^. All samples were measured five-fold and the spectrum baseline was corrected. Spectragryph 1.2 (F. Menges, Oberstdorf, Germany) [32] was used to average the five measurements and to normalize to a height of 1.0 (resulting in the final spectrum) at 1018 cm^−1^ for the samples with a high galacturonic acid contend (OXO 0.4 and 1.0), at 1038 cm^−1^ for the low galacturonic acid content samples (OXO N and OXO 0.2), and at 1042 cm^−1^ for the mucilage (OXO M). The second derivative of the averaged spectra was calculated using the same software. 

### 3.11. Atomic Force Microscopy Imaging

All the lyophilized mucilage fractions obtained by IEC were dialyzed using ZelluTrans/Roth^®^ dialysis membranes (Carl Roth GmbH & Co. KG, Karlsruhe, Germany; MWCO 3500) and were freeze-dried again. 0.1 mg·mL^−1^ solutions of each polysaccharide were prepared, and 20 μL was dropped onto freshly cleaved mica and spread by spin coater SPIN150i (SPS-EUROPE, Putten, The Netherlands). Next, the specimen was dried in a desiccator (relative humidity 15%) at 22 °C overnight before the AFM studies. The studies were executed in ambient air at room temperature and 25–30% relative humidity. At least ten images were collected for each sample.

AFM height images were executed using Multimode 8 with a Nanoscope V controller (Bruker, Billerica, MA, USA) using the semiautomatic high-speed tapping mode. A silicon nitride cantilever with a 2 nm-nominal radius pyramidal tip, a nominal resonance frequency of 130 kHz and a nominal spring constant of 0.4 N/m (ScanAsyst Air-HR, Bruker, Billerica, MA, USA) was applied. The following scan settings were established: scan size 2 × 2 μm, 5 × 5 μm, 10 × 10 μm resolution 512 × 512 points, and scan rate 2.0 Hz. 

#### Image Analysis

AFM height images were processed and analyzed using SPIP 6.2.0 software (Image Metrology, Hørsholm, Denmark). Prior to image analysis, all of the images were flattened using a third-order polynomial fitting and global bow removal and then filtered using a standardized roughness filter and noise reduction procedure and smoothed by Gaussian filtering. The height and length of macromolecules were determined using the ‘Particle and Pore Analysis’ module of SPIP software [33]. For all AFM images a set of pixel thresholds was applied, which included cutting out elements lower than 20 pm in the Z direction and the segmentation of particles if the minima between them were below 10 pm. Finally, the skeletonized images were used for the determination of length and height of particles.

The following parameters (as defined by SPIP 6.2.0 software) were calculated: fiber length, as the longest path in the skeleton; skeleton length, as the sum of the length of all branches (or segments) of the skeleton; aspect ratio, defined as length over breadth; and elongation, as the measure indicating how elongated a shape is. A square or circle will return the value zero. As these shapes change towards a long rectangle or ellipse, the returned value converges towards 1.0. Elongation = (length − breadth)/length

### 3.12. Evaluation of the Prebiotic Potential

The prebiotic potential was evaluated by monitoring the growth curves of the selected bacterial strains as described in [15], the modification being that all work was performed under anaerobic conditions (85% N_2_, 10% CO_2_, 5% H_2_). *B. galacturonicus* was the sole exception, as it cannot use glucose as a carbon source, which was cultivated following the instructions and media (DSMZ 1265) of the supplier. The species were selected based on their known (or potential) probiotic characteristics [34]. Shortly, the bacteria were cultivated in an anaerobic tent in the appropriate media supplemented with glucose or galacturonic acid and incubated at 37 °C. After overnight growth, the cultures were washed, diluted to a starting optical density at 600 nm (OD_600_) of 0.1, and dispensed into sterile 96-well flat-bottomed microplates (Corning Inc., Corning, NY, USA). The well was filled 1:1 with the culture and with the appropriate supplemented media to a final concentration of 1%*_w_*_/*v*_ of the polysaccharide to be tested. The OD_600_ was recorded every 30 min at 37 °C for a total of 48 h using a plate reader (Multiskan™ GO Microplate Spectrophotometer, Thermo Fisher Scientific, Waltham, MA, USA) located inside the anaerobic tent. Bacteria plus media without polysaccharide was used as the negative control (NA). All experiments were performed in duplicate. All reagents were introduced into the anaerobic tent at least the day before the experiment to assure that they were in an anaerobic condition. Fraction 1.0 M was not tested with *B. thetaiotaomicron* due to a lack of starting material.

## 4. Conclusions

This work has shown that the mucilage extracted from the cladodes of *O. joconostle* is a macromolecular assembly composed of a variety of quite structurally complex polysaccharides, held together by covalent and non-covalent bonds. Of these polysaccharides, one is composed only of neutral monosaccharides, while the three others include galacturonic acid in differing proportions. Given the different structural features (i.e., monosaccharide composition, molecular dimensions, and linkage types) present in each polysaccharide population, different representative commensals gut bacteria are able to selectively depolymerize and ferment them. This specificity, coupled with their resistance to the acidic conditions of the upper gastrointestinal tract, makes these polysaccharides attractive candidates as prebiotics. They most likely reach the large intestine intact, where they will increase the numbers of beneficial saccharolytic bacteria. Furthermore, the oligosaccharides resulting from the degradation of the polysaccharides can be then utilized by members of *Lactobacilli* and *Bifidobacteria*. 

The high biomass productivity of *Opuntia* spp. in arid lands, which are not suitable for most other crops, in combination with the presence of these highly complex polysaccharides, offers significant economic potential. Uses include forage for animals, production of precise prebiotic and synbiotic formulations, or a source of raw material for the concept of a green refinery. In addition, these results, and the methodology used to reach them, are a tool to evaluate the changes in phenotype, helping to guide the breeding strategy.

## Figures and Tables

**Table 1:** Molecular dimensions of *O. joconostle* mucilage (extracted from the cladodes) and its composing polysaccharides found after ion exchange chromatography fractionation.

Sample	Yield	${M_{w}^{-}}^{\;1}$	${M_{n}^{-}}^{\;2}$	*Ð* ^3^	Distribution
	%_*w*/*w*_	(g∙mol^−1^)	(g∙mol^−1^)		(g∙mol^−1^)
OXO M [14]	100.0	4.5 × 10^5^	1.3 × 10^5^	3.40	5.5 × 10^3^–1.3 × 10^6^
OXO N	8.8	2.1 × 10^5^	7.9 × 10^4^	2.68	2.5 × 10^3^–7.4 × 10^5^
OXO 0.2	44.8	2.8 × 10^5^	1.1 × 10^5^	2.41	3.0 × 10^3^–8.3 × 10^5^
OXO 0.4	36.8	1.1 × 10^5^	3.9 × 10^4^	2.86	9.3 × 10^2^–6.8 × 10^3^
OXO 1.0	6.4	2.2 × 10^5^	5.7 × 10^4^	3.77	1.2 × 10^3^–7.9 × 10^5^

^1^ average mass molar mass. ^2^ average number molar mass. ^3^ dispersity, *Ð* = $M_{w}^{-}/M_{n}^{-}$.

**Table 2:** Monosaccharide composition and total carbohydrate content of the heteropolysaccharides that compose the mucilage extracted from the cladodes of *O. joconostle*.

Sample	Rha	Ara	Xyl	Gal	Fuc	Glc	GalA	Carbohydrate Content
	(%_mol_) ^1^	(%_mol_) ^1^	(%_mol_) ^1^	(%_mol_) ^1^	(%_mol_) ^1^	(%_mol_) ^1^	(%_mol_) ^1^	(%)
OXO M [14]	3.4 ± 0.2	28.4 ± 0.5	16.7 ± 1.7	33.6 ± 0.2	n.d.	2.3 ± 0.5	15.5 ± 1.7	81.2 ± 0.1
OXO N	4.3 ± 0.1	18.6 ± 0.6	10.6 ± 0.3	62.7 ± 2.8	1.8 ± 0.0	1.9 ± 0.5	n.d.	93.5 ± 0.1
OXO 0.2	8.3 ± 0.2	33.6 ± 0.9	20.1 ± 0.4	27.1 ± 1.9	0.8 ± 0.1	0.7 ± 0.1	9.6 ± 0.1	91.8 ± 0.1
OXO 0.4	8.3 ± 0.5	26.5 ± 1.0	13.4 ± 0.2	25.3 ± 3.2	1.4 ± 0.4	0.5 ± 0.2	24.6 ± 0.1	92.1 ± 0.1
OXO 1.0	8.3 ± 0.4	23.0 ± 3.0	11.9 ± 0.5	20.5 ± 1.2	0.6 ± 0.4	1.1 ± 0.4	34.6 ± 0.6	91.3 ± 0.1
OXO M ^2^	7.9	28.8	16.1	29.2	1.1	0.8	16.1	

^1^ Confidence interval calculated at 95% (*n* = 4). ^2^ Recreated compositions, calculated from the monosaccharide content and yield for each fraction. n.d., not detected.

## Data Availability

Not applicable.

## Appendix A

**Table A1:** Polysaccharides’ fiber and aggregates parameters as calculated by the imaging software.

**Sample**	**Fibers**			
	**Fiber Length**	**Skeleton Length**	**Mean Height**	**Elongation**
	**(nm)**	**(nm)**	**(nm)**	
OXO M	546 ± 27.8	761 ± 67.7	0.33 ± 0.0	0.59 ± 0.1
OXO N	365 ± 7.6	554 ± 30.1	0.44 ± 0.0	0.47 ± 0.0
OXO 0.2	436 ± 0.2	564 ± 21.6	0.40 ± 0.0	0.52 ± 0.0
OXO 0.4	438 ± 11.6	773 ± 39.7	0.41 ± 0.0	0.43 ± 0.0
OXO 1.0	423 ± 20.6	1062 ± 157.9	0.45 ± 0.0	0.48 ± 0.0
	**Aggregates**			
	**Mean Height**	**Perimeter**	**Area**	**Volume**
	**(nm)**	**(nm)**	**(nm^2^)**	**(nm^3^)**
OXO M	0.80 ± 0.0	195.4 ± 2.7	3353 ± 3598	5752 ± 1713
OXO N	n.a.	n.a.	n.a.	n.a.
OXO 0.2	0.42 ± 0.0	208.7 ± 2.2	3948 ± 103	3382 ± 336
OXO 0.4	n.a	n.a	n.a	n.a
OXO 1.0	0.49 ± 0.0	188.5 ± 2.5	3503 ± 3738	2785 ± 335

## References

[1] Yang X., Baskin J.M., Baskin C.C., Huang Z. (2012). More than Just a Coating: Ecological Importance, Taxonomic Occurrence and Phylogenetic Relationships of Seed Coat Mucilage. Perspect. Plant Ecol. Evol. Syst..

[2] Gregory M., Baas P. (1989). A Survey of Mucilage Cells in Vegetative Organs of the Dicotyledons. Isr. J. Bot..

[3] Galloway A.F., Knox P., Krause K. (2020). Sticky Mucilages and Exudates of Plants: Putative Microenvironmental Design Elements with Biotechnological Value. New Phytol..

[4] Bredenkamp C.L., van Wyk A.E. (1999). Structure of Mucilaginous Epidermal Cell Walls in *Passerina* (Thymelaeaceae). Bot. J. Linn. Soc..

[5] Mastroberti A.A., de Araujo Mariath J.E. (2008). Development of Mucilage Cells of *Araucaria angustifolia* (Araucariaceae). Protoplasma.

[6] Hashem H.A., Mohamed A.H., Hasanuzzaman M. (2020). Strategies for Drought Tolerance in Xerophytes. Plant Ecophysiology and Adaptation under Climate Change: Mechanisms and Perspectives I: General Consequences and Plant Responses.

[7] Zhang R., Chen Y., Yang L., Xie Y., Yan W., He Z. (2020). Study on the Relationship between Mucilage Cells and Low Temperature Tolerance of *Sedum Aizoon* L.. IOP Conf. Ser. Earth Environ. Sci..

[8] Orozco-Segovia A., Márquez-Guzmán J., Sánchez-Coronado M.E., Gamboa de Buen A., Baskin J.M., Baskin C.C. (2007). Seed Anatomy and Water Uptake in Relation to Seed Dormancy in *Opuntia tomentosa* (Cactaceae, Opuntioideae). Ann. Bot..

[9] Trachtenberg S., Fahn A. (1981). The Mucilage Cells of *Opuntia ficus-indica* (L.) Mill.-Development, Ultrastructure, and Mucilage Secretion. Bot. Gaz..

[10] Trachtenberg S., Mayer A.M. (1981). Calcium Oxalate Crystals in *Opuntia ficus indica* (L.) Mill.: Development and Relation to Mucilage Cells—A Stereological Analysis. Protoplasma.

[11] Peña-Valdivia C.B., Trejo C., Arroyo-Peña V.B., Sánchez Urdaneta A.B., Balois Morales R. (2012). Diversity of Unavailable Polysaccharides and Dietary Fiber in Domesticated Nopalito and Cactus Pear Fruit (*Opuntia* Spp.). Chem. Biodivers..

[12] Astello-García M.G., Cervantes I., Nair V., Santos-Díaz M.d.S., Reyes-Agüero A., Guéraud F., Negre-Salvayre A., Rossignol M., Cisneros-Zevallos L., Barba de la Rosa A.P. (2015). Chemical Composition and Phenolic Compounds Profile of Cladodes from *Opuntia* Spp. Cultivars with Different Domestication Gradient. J. Food Compos. Anal..

[13] López-Palacios C., Peña-Valdivia C.B., Reyes-Agüero J.A., Rodríguez-Hernández A.I. (2012). Effects of Domestication on Structural Polysaccharides and Dietary Fiber in Nopalitos (*Opuntia* Spp.). Genet. Resour. Crop Evol..

[14] Cruz-Rubio J.M., Mueller M., Loeppert R., Viernstein H., Praznik W. (2020). The Effect of Cladode Drying Techniques on the Prebiotic Potential and Molecular Characteristics of the Mucilage Extracted from *Opuntia ficus-indica* and *Opuntia joconostle*. Sci. Pharm..

[15] Cruz-Rubio J.M., Mueller M., Viernstein H., Loeppert R., Praznik W. (2021). Prebiotic Potential and Chemical Characterization of the Poly and Oligosaccharides Present in the Mucilage of *Opuntia ficus-indica* and *Opuntia joconostle*. Food Chem..

[16] Guevara-Arauza J.C., de Jesús Ornelas-Paz J., Pimentel-González D.J., Rosales Mendoza S., Soria Guerra R.E., Paz Maldonado L.M.T. (2012). Prebiotic Effect of Mucilage and Pectic-Derived Oligosaccharides from Nopal (*Opuntia ficus-indica*). Food Sci. Biotechnol..

[17] Szymanska-Chargot M., Zdunek A. (2013). Use of FT-IR Spectra and PCA to the Bulk Characterization of Cell Wall Residues of Fruits and Vegetables Along a Fraction Process. Food Biophys..

[18] Kacčuráková M., Capek P., Sasinková V., Wellner N., Ebringerová A. (2000). FT-IR Study of Plant Cell Wall Model Compounds: Pectic Polysaccharides and Hemicelluloses. Carbohydr. Polym..

[19] Sengkhamparn N., Bakx E.J., Verhoef R., Schols H.A., Sajjaanantakul T., Voragen A.G.J. (2009). Okra Pectin Contains an Unusual Substitution of Its Rhamnosyl Residues with Acetyl and Alpha-Linked Galactosyl Groups. Carbohydr. Res..

[20] Gnanasambandam R., Proctor A. (2000). Determination of Pectin Degree of Esterification by Diffuse Reflectance Fourier Transform Infrared Spectroscopy. Food Chem..

[21] Célino A., Gonçalves O., Jacquemin F., Fréour S. (2014). Qualitative and Quantitative Assessment of Water Sorption in Natural Fibres Using ATR-FTIR Spectroscopy. Carbohydr. Polym..

[22] Palacio S., Aitkenhead M., Escudero A., Montserrat-Martí G., Maestro M., Robertson A.H.J. (2014). Gypsophile Chemistry Unveiled: Fourier Transform Infrared (FTIR) Spectroscopy Provides New Insight into Plant Adaptations to Gypsum Soils. PLoS ONE.

[23] Kaeswurm J.A.H., Scharinger A., Teipel J., Buchweitz M. (2021). Absorption Coefficients of Phenolic Structures in Different Solvents Routinely Used for Experiments. Molecules.

[24] Canteri M.H.G., Renard C.M.G.C., Le Bourvellec C., Bureau S. (2019). ATR-FTIR Spectroscopy to Determine Cell Wall Composition: Application on a Large Diversity of Fruits and Vegetables. Carbohydr. Polym..

[25] Liu X., Renard C.M.G.C., Bureau S., Le Bourvellec C. (2021). Revisiting the Contribution of ATR-FTIR Spectroscopy to Characterize Plant Cell Wall Polysaccharides. Carbohydr. Polym..

[26] Sene C.F.B., McCann M.C., Wilson R.H., Grinter R. (1994). Fourier-Transform Raman and Fourier-Transform Infrared Spectroscopy (An Investigation of Five Higher Plant Cell Walls and Their Components). Plant Physiol..

[27] Ravcheev D.A., Godzik A., Osterman A.L., Rodionov D.A. (2013). Polysaccharides Utilization in Human Gut Bacterium *Bacteroides Thetaiotaomicron*: Comparative Genomics Reconstruction of Metabolic and Regulatory Networks. BMC Genom..

[28] Centanni M., Hutchison J.C., Carnachan S.M., Daines A.M., Kelly W.J., Tannock G.W., Sims I.M. (2017). Differential Growth of Bowel Commensal Bacteroides Species on Plant Xylans of Differing Structural Complexity. Carbohydr. Polym..

[29] Misra S., Pandey P., Mishra H.N. (2021). Novel Approaches for Co-Encapsulation of Probiotic Bacteria with Bioactive Compounds, Their Health Benefits and Functional Food Product Development: A Review. Trends Food Sci. Technol..

[30] Riva A., Kolimár D., Spittler A., Wisgrill L., Herbold C.W., Abrankó L., Berry D. (2020). Conversion of Rutin, a Prevalent Dietary Flavonol, by the Human Gut Microbiota. Front. Microbiol..

[31] Hazra A. (2017). Using the Confidence Interval Confidently. J. Thorac. Dis..

[32] Menges F. (2022). Spectragryph—Optical Spectroscopy Software. http://www.effemm2.de/spectragryph/.

[33] Cybulska J., Halaj M., Cepák V., Lukavský J., Capek P. (2016). Nanostructure Features of Microalgae Biopolymer. Starch-Stärke.

[34] Khalighi A., Behdani R., Kouhestani S., Khalighi A., Behdani R., Kouhestani S. (2016). Probiotics: A Comprehensive Review of Their Classification, Mode of Action and Role in Human Nutrition.

